# Rates and predictors of data and code sharing in the medical and health sciences: Protocol for a systematic review and individual participant data meta-analysis.

**DOI:** 10.12688/f1000research.53874.2

**Published:** 2021-09-09

**Authors:** Daniel G. Hamilton, Hannah Fraser, Fiona Fidler, Steve McDonald, Anisa Rowhani-Farid, Kyungwan Hong, Matthew J. Page

**Affiliations:** 1 MetaMelb Research Group, School of BioSciences, University of Melbourne, Parkville, Victoria, 3010, Australia; 2 School of Historical and Philosophical Studies, University of Melbourne, Parkville, Victoria, 3010, Australia; 3 School of Public Health & Preventive Medicine, Monash University, Melbourne, Victoria, 3004, Australia; 4 Department of Pharmaceutical Health Services Research, University of Maryland, Baltimore, Maryland, 21201, USA

**Keywords:** Systematic review, Meta-analysis, Data sharing, Code sharing, Medicine, Health sciences

## Abstract

Numerous studies have demonstrated low but increasing rates of data and code sharing within medical and health research disciplines. However, it remains unclear how commonly data and code are shared across all fields of medical and health research, as well as whether sharing rates are positively associated with implementation of progressive policies by publishers and funders, or growing expectations from the medical and health research community at large. Therefore this systematic review aims to synthesise the findings of medical and health science studies that have empirically investigated the prevalence of data or code sharing, or both. Objectives include the investigation of: (i) the prevalence of public sharing of research data and code alongside published articles (including preprints), (ii) the prevalence of private sharing of research data and code in response to reasonable requests, and (iii) factors associated with the sharing of either research output (e.g., the year published, the publisher’s policy on sharing, the presence of a data or code availability statement). It is hoped that the results will provide some insight into how often research data and code are shared publicly and privately, how this has changed over time, and how effective some measures such as the institution of data sharing policies and data availability statements have been in motivating researchers to share their underlying data and code.

## Background

Over the last two decades there has been growing calls on the scientific community to improve the transparency of many elements of the scholarly research lifecycle. One key aspect that is of interest to this movement - often termed the “open science” movement - includes improving access to both the raw data underlying published research findings, as well as the syntax from relevant statistical software used to generate them (“research code”). ^1^^,^^2^

While open science principles are being increasingly adopted and promoted by major medical and health research stakeholders, the debate about the advantages, disadvantages, ethics, and legalities of sharing research data alongside published research is far from settled. For example, from one perspective greater availability of research data and code is considered a desirable goal as it allows for independent verification of findings, greater detection of errors, and is associated with increased scholarly impact metrics. ^2^^–^^4^ Sharing data also facilitates more efficient and comprehensive aggregation of existing research findings, testing of secondary hypotheses not considered by the original authors, as well as evaluation of the robustness of chosen analytic strategies. ^3^^,^^5^^,^^6^ However, in contrast, other research points to many barriers to sharing data, such as: the navigation of participant privacy concerns, proprietary data and licensing terms, a lack of incentives to share, fears among researchers concerning loss of recognition and control over the research outputs (i.e., right to publish) and the misuse or misinterpretation of shared data, as well as the time and resource burdens associated with archiving data in a way that enables reuse. ^7^^–^^11^

Ultimately, despite contrasting evidence and opinions on the topic, funders of medical and health research continue to institute increasingly progressive policies governing sharing of research data and code. For example, the National Institutes of Health (NIH) and the National Science Foundation (NSF) both require grant applicants to submit comprehensive data management plans, ^9^ with the National Institutes of Health also expecting NIH-funded researchers to share data generated from large-scale human or non-human genomic research. ^12^ Similarly, publishers of medical research are also adopting more progressive data and code sharing policies. For example, a recent small survey of medical journal editors in 2019 by the first author observed 15% and 10% have instituted policies requiring public deposition of data and code sharing, respectively. ^13^ The same study also noted that 28% of medical journals required authors to include a formalised data availability statement, ^13^ which is also now stipulated by the International Committee of Medical Journal Editors’ (ICMJE) clinical trial data sharing policy for articles reporting the findings of clinical trials. ^14^

To date, numerous studies have investigated how prevalent data and code sharing is. With regard to medicine and health, this research has reported traditionally low, but increasing rates of sharing and use of data availability statements across many fields, including but not limited to: biomedicine, ^15^^–^^18^ cardiology, ^19^ oncology, ^20^ orthopaedics, ^21^ otolaryngology, ^22^ radiology ^23^ and COVID-19-related research. ^24^ Previous research has also highlighted low sharing of clinical trial data both publicly, ^25^ as well as in response to reasonable private requests (e.g. for a meta-analysis, secondary analysis, sample size calculation). ^26^^–^^28^ However, how common sharing of data and code is across all medical and health research, how this has changed over time, as well as how strongly it is influenced by journal and funder policymaking and community expectations - particularly in light of the COVID-19 pandemic ^29^ - remains unclear.

The aim of this review is therefore to summarise the characteristics and synthesise the findings of this research to provide some insight into how well some of these policies are working at increasing sharing of data and code. It is hoped that the results will be able to provide some insights into how often research data and code are shared publicly and privately, how this has changed over time, and how effective some measures, such as the institution of mandatory data sharing policies and data availability statements have been in motivating researchers to share.

## Objectives

To summarise the characteristics and synthesise the findings of research that has empirically investigated (i) the prevalence of public sharing of research data and code alongside published articles (including preprints), (ii) the prevalence of private sharing of research data and code in response to reasonable requests, and (iii) factors associated with the sharing of either research output (e.g., the year published, the publisher’s policy on sharing, the presence of a data or code availability statement).

## Methods

This protocol was developed in accordance with the PRISMA-P, ^30^ PRISMA 2020 ^31^ and PRISMA-IPD ^5^ statements and was pre-registered on May 28th, 2021 on the Open Science Framework ( https://osf.io/7sx8u). Since this review will only collect and analyse data derived from published articles, ethical review and approval was not sought.

### Criteria for considering studies for this review

**Types of studies**

This review will include studies that have empirically investigated the prevalence of data or code sharing, or both (termed “meta-research studies”), among a sample of scientific articles presenting original research from the medical and health sciences (termed “primary studies”). Studies can be published or unpublished articles (e.g., preprints) of any format (e.g., full-text article, conference abstract, research letter).

We will include meta-research studies regardless of (i) whether they have sampled primary studies in a random or non-random fashion, (ii) how much of a primary study’s data has been shared (e.g., partial sharing versus full sharing), (iii) the types of data considered for sharing (e.g., microarray data, genomic data, macromolecular data, imaging data, clinical data, simulated data, synthetic data) or (iv) whether the availability of data and code has been verified by the authors of the meta-research study. However, we will exclude any meta-research studies that investigated data or code availability (i) as part of a single individual participant data (IPD) meta-analysis, (ii) for a single primary study (i.e., case report) or (iii) via other forms of research dissemination (e.g., clinical trial registry entries, data repository pages).

**Types of data**

Three types of data will be of interest to this review – aggregate data (i) reported by included meta-research studies, (ii) derived from available IPD or (iii) provided on request from meta-research study authors.

For all eligible meta-research studies, reported summary statistics relating to (i) demographic variables of the primary studies, (ii) estimates of the prevalence of data or code sharing (publicly or privately) for the relevant sample of primary articles, and (iii) estimates of the association between data or code sharing (publicly or privately) and demographic variables of interest will be collected. Refer to the Data extraction and management section for further details about the specific variables of interest to the study.

If meta-research studies use differing definitions to those outlined in this protocol (e.g., consider “available on request” declarations as “shared”), we will only extract findings compliant with our protocol, or recode variables in line with definitions outlined in this protocol when possible. Similarly, if meta-research studies report relevant outcome measures in aggregate (e.g., report results for a mixture of medical and non-medical disciplines, or across an extended period of publication dates), we will only extract findings conforming to variables of interest outlined in the protocol (e.g., prevalence rates among medical and health research, prevalence rates by eligible year(s) of publication).

For studies where the above required information has been collected, but is not reported in the published article, publicly available IPD will be used to derive summary statistics of interest, such as: prevalence rates for our primary outcome measures, or risk ratios for our secondary outcome measures (see Types of outcome measures) and proposed subgroup analyses (see Subgroup analysis and investigation of heterogeneity). If IPD are not available publicly, we will request them from corresponding authors, or if authors are unwilling or unable to share IPD, they will be asked to provide the required summary statistics.

If none of the three types of data can be obtained, results will be included in the qualitative analysis (e.g., tabulated and narratively discussed), and in any relevant forest plots, but not included in the statistical synthesis. However, given the nature of the studies under review (i.e., studies investigating data and code availability among publicly available articles), and following pilot literature searching, it is expected that most of the authors of meta-research studies will have either already publicly shared IPD, or would be receptive and able to do so.

**Types of methods**

There are four types of data and code sharing that will be examined as part of this review:

1. Declarations by primary authors that the research data and code has been made publicly available (reported public availability).
2. Confirmation that research data and code has been made publicly available following independent interrogation of author declarations, and verification of availability (actual public availability).
3. Declarations by primary authors that the research data and code are available upon request (reported private availability).
4. Confirmation that research data and code are available in response to a private request (actual private availability).

‘Public sharing’ will be broadly construed as the deposition of research data or code into a theoretically publicly accessible location (e.g., a freely accessible data repository, or an article’s supplementary material). For primary studies reporting data as “available on request”, this will be considered as privately available. Furthermore, if not explicitly verified by the meta-research study’s authors as available, it will be assumed that reported public sharing estimates represent ‘reported availability’. It should also be noted that ‘sharing’ in the context of this review will be defined as the sharing of data or code required to theoretically verify or reconstruct at least one of the primary study’s published findings.

**Types of outcome measures**

We will include four primary outcome measures for research data and code respectively:

Research data

1. Prevalence of studies in which authors declare their data is publicly available (reported public availability).
2. Prevalence of studies in which meta-researchers confirm study data is publicly available following independent interrogation of author declarations, and verification of availability (actual public availability).
3. Prevalence of studies in which authors declare their data is privately available (e.g. “available on request” statements) (reported private availability).
4. Prevalence of studies in which meta-researcher confirm study data was released in response to a private request (actual private availability).

Research code

1. Prevalence of studies in which authors declare their code is publicly available (reported public availability).
2. Prevalence of studies in which meta-researchers confirm study code is publicly available following independent interrogation of author declarations, and verification of availability (actual public availability).
3. Prevalence of studies in which authors declare their code is privately available (e.g. “available on request” statements) (reported private availability).
4. Prevalence of studies in which meta-researcher confirm study code was released in response to a private request (actual private availability).

We will also include seven secondary outcome measures:

1. The prevalence of data availability statements in study reports.
2. The prevalence of code availability statements in study reports.
3. The association between the presence of a data availability statement and public sharing of research data (reported or actual availability), for example, does requiring a data availability statement increase the likelihood of sharing data.
4. The association between the presence of a code availability statement and public sharing of research code (reported or actual availability), for example, does requiring a code availability statement increase the likelihood of sharing code.
5. The association between a journal’s policy on data sharing (any ‘mandatory posting’ policy versus other policy) and public sharing of research data (reported or actual availability).
6. The association between the journal’s policy on data sharing (‘make available on request’ policy versus other non-mandatory policy) and private sharing of research data (reported or actual availability).
7. The association between public sharing of research data (reported or actual availability) and the sharing of code (reported or actual availability).

### Search methods for identification of studies

**Electronic searches**

We will search the following bibliographic databases and preprint servers from inception for relevant meta-research studies:

• Ovid MEDLINE
• Ovid Embase
• medRxiv
• bioRxiv
• MetaArXiv

The search was developed by an information specialist (SM) using a sample of 14 papers deemed relevant to the topic. The search strategy was designed in Ovid MEDLINE and initially tested on a subset of the 14 papers and then iteratively refined to ensure that all papers were retrieved by the search. An analysis of the Medical Subject Headings (MeSH) applied to these 14 papers revealed several potentially relevant terms (e.g., Reproducibility of Results, Information Dissemination) but none were considered appropriate to include in the strategy because they lacked precision. The same search was applied to Ovid Embase, allowing for modifications to the search syntax. The Ovid MEDLINE search syntax was then adapted by the first author into Lucene search syntax to search MetaArXiv, and R programming language to search the medRxiv and bioRxiv preprint servers via the medrxivr package. ^32^ No restrictions will be placed on any of the searches with regard to language of publication. The search strategies for each database are available on the Open Science Framework ( https://osf.io/h75v4/).

### Searching other resources

The team will screen reference lists of relevant studies identified by the search, as well as the bibliographies of all included studies. We will also conduct forward citation searches of included articles, as well as browse other preprint servers (PeerJ, Research Square) and online resources (Open Science Framework, aspredicted.org and connectedpapers.com) to help identify further published, unpublished and pre-registered studies.

### Data collection and analysis

**Selection of studies**

Results from all searches will be imported into Covidence (Covidence systematic review software, Veritas Health Innovation, Melbourne, Australia) and deduplicated. For the results of the preprint server searches, if published versions of preprints are available, they will be sourced and screened for eligibility, if not we will select and screen the preprint. All titles and abstracts identified by the search strategies above will be independently screened against the eligibility criteria by two authors in parallel. Following title and abstract screening, two authors will independently assess full-text articles (where available) for inclusion. We will attempt to translate foreign-language articles flagged as potentially eligible using Google Translate or native speakers known to the team. If unable to translate the document successfully, we will exclude the study. All disagreements on the eligibility of studies at each phase will be resolved via discussion, or a third author if required. We will prepare a flow diagram in accordance with both the PRISMA 2020 statement and PRISMA-IPD extension outlining the flow of identified articles throughout each stage of the review. ^5^^,^^31^ The reasons for exclusion of full-text articles will also be documented.

### Data extraction and management

**Summary statistics derived from meta-research studies**

Once the list of included articles is determined, two authors will independently extract summary statistics from each included meta-research study using a predefined data extraction form developed for this review. Any differences in coding will be resolved via discussion, or a third author if consensus cannot be reached. The data extraction form will be pilot tested by the data extractors on at least five randomly selected included articles, and if required, modified prior to use.

The following key variables will be extracted from included articles:

• Characteristics of the meta-research study, including but not limited to: study title, DOI, journal, publication date, health/medical discipline(s) of interest, the number of primary studies examined (sample size), sampling strategy, protocol availability, data availability and so on;
• Data on estimates of prevalence as outlined in Types of outcome measures;
• Data on factors associated with sharing as outlined in Types of outcome measures.

A comprehensive list of the variables to be extracted is available on the Open Science Framework ( https://osf.io/h75v4/).

**Summary statistics derived from individual participant data**

Demographic variables and outcome measures of relevance to the study that are not reported by meta-research studies but appear to have been collected by study authors will be investigated further. If the underlying IPD and data dictionary from the meta-research study are publicly available, one author (DGH) will calculate the desired information from the raw data and enter it into a pre-prepared CSV-formatted spreadsheet. If IPD are not available, the corresponding author of the meta-research study will be contacted to request the required information or the raw data. A comprehensive list of the variables that may be extracted from available IPD is available on the Open Science Framework ( https://osf.io/h75v4/).

### Assessment of the risk of bias in included studies

The following criteria have been created with guidance from previous Cochrane Methodology Reviews ( Table 1). ^33^^,^^34^ Two authors will independently classify each included study, with any differences in coding resolved via discussion, or a third author if consensus cannot be reached. We will contact authors of included studies for additional information when assessments are initially classified as unclear.

**Table 1.** Risk of bias criteria.

Item	Low risk of bias	High risk of bias	Unclear risk of bias
Risk of sampling bias	The meta-research study evaluated a random sample of primary articles.	The meta-research study included a non- or pseudo-random sample of primary articles.	The sampling frame for the sample of primary articles is unclear.
Risk of selective reporting bias	Eligible outcomes and associations reported in the protocol for the meta-research study are fully reported in the results section of the publication.	Not all eligible outcomes and associations reported in the protocol for the meta-research study are reported in the results section of the publication.	It is unclear if all eligible outcomes and associations are fully reported in the results section of the publication (e.g., because a study protocol for the meta-research study is unavailable).
Risk of article selection bias	Details about which studies were excluded from the study and why have been shared and match the criteria described in the methods.	Details about which studies were excluded and why were not reported.	Details about the eligibility criteria and study selection process is unclear.
Risk of errors in the accuracy of reported estimates	All outcome data were either: manually coded by at least two people independently in parallel or coded by one person and checked in full by another.	Outcome data was manually coded by: only one researcher, only an automated algorithm, or according to another methodology different from that outlined in the Low Risk category.	The method used to extract data from the included primary studies is unclear.

Given the aim to differentiate between studies with higher and lower risk of bias, a study will be deemed as having a low risk of bias if all the above criteria are assessed as low risk of bias, and high risk of bias if any one criterion is assessed as high or unclear risk of bias.

### Measures of the effect of the methods

For studies that report estimates of the prevalence of data or code sharing, we will report percentages (no. of articles that shared/no. of relevant articles assessed) and 95% confidence intervals (CI) calculated using the Wilson score interval method. ^35^ The measures of the prevalence and association between a factor and data sharing will be dependent on the summary statistics used and reported by the authors of the meta-research studies, and the availability of IPD. For studies that have investigated the association between relevant factors and the sharing of research data (refer to Types of outcome measures for more information), we will report risk ratios with 95% confidence intervals. We will standardise our reporting so that risk ratios greater than one will indicate a higher likelihood of data availability. If authors of meta-research studies report odds ratios instead of risk ratios we will convert them to risk ratios using the formula proposed by Grant. ^36^ Where studies do not report this information, prevalence rates and risk ratios will be calculated from the raw data if it is available, or requested from the corresponding author.

### Unit of analysis issues

It is possible that there may be some overlap in the primary articles examined across included meta-research studies. Once the list of included studies is finalised, we will check for potential overlap by comparing reported primary article characteristics across meta-research studies (e.g., discipline(s) of interest, publication dates, publication outlets, study designs). The team will assess the degree of overlap and flag studies for which the likelihood of overlap is high, and then will check the IPD of flagged studies for duplicate primary articles by interrogating unique identifiers across datasets from included studies (e.g., DOI, PMID, study title). We will report whether this issue was able to be addressed, and if not, its likelihood of occurring and the likely impact on the findings of the review.

### Dealing with missing data

For eligible studies where raw data are unavailable and information on study characteristics (e.g., methods for identifying and selecting primary articles) or outcomes (e.g., prevalence of sharing by a subgroup of interest) is missing, corresponding authors will be contacted. If the required information cannot be retrieved, available information will be discussed narratively. We will not impute missing data using statistical techniques. We will instead discuss missing data narratively.

### Assessment of heterogeneity

We will assess the similarity/dissimilarity of methodological aspects of included studies, particularly with respect to definitions of “data”, “code” and “sharing”. We will evaluate statistical heterogeneity by inspecting the distribution of effects within forest plots and the magnitude of corresponding I ^2^ statistics and their 95% confidence intervals. ^37^ We will also further evaluate statistical heterogeneity by calculating prediction intervals for our primary outcomes where more than four studies are included. ^38^ Prediction intervals estimate the likely range of effect sizes (prevalence rates and risk ratios) that could be expected across similar studies. ^39^^,^^40^ Prediction intervals will be calculated in R using the meta package ^41^ which implements the formula proposed by Higgins and colleagues ^42^ (equation 12).

### Assessment of reporting biases

We believe that the likelihood of this review being affected by publication bias is low given studies of interest to the review appear to be mostly exploratory in nature, with a focus on reporting prevalence rates rather than testing specific hypotheses. However, we will assess the risk of publication bias by searching for pre-registered protocols of eligible meta-research studies. We will also assess the risk of selective-reporting bias by comparing what authors of meta-research studies reported, with what they stated in the protocol for the study (see Assessment of the risk of bias in included studies).

### Data synthesis

This review will adopt a “two stage” approach to IPD meta-analysis, whereby we will examine meta-research studies in the first stage to extract summary statistics, or retrieve them from available IPD or from corresponding authors. Where data are available and appropriate (e.g., low heterogeneity), in the second stage, results from meta-research studies will then be pooled as per conventional meta-analysis. ^43^ For each of the primary and secondary outcome measures, we will pool prevalence and risk ratio estimates using a random-effects model and will calculate 95% CIs for the summary effect using the method developed by Hartung-Knapp-Sidik-Jonkman. ^44^ Prevalence rates will be transformed using the Freeman-Tukey double arcsine transformation and combined using standard inverse variance methods. ^45^ When it is not possible to meta-analyse due to clinical and/or statistical heterogeneity we will report prevalence, risk ratios, 95% CIs and p-values in tables.

### Subgroup analysis and investigation of heterogeneity

Where data are available, we will perform subgroup analyses to investigate whether the prevalence of public sharing of data is associated with the following factors:

• Whether primary studies were defined by the study authors as a clinical trial (any phase) or not;
• Whether primary studies studied COVID-19 or not;
• Whether primary studies directly studied, or used any data derived from, human participants or not;
• Whether primary studies were subject to any mandatory sharing policies by the funders of the study or not;
• Whether primary studies posted a preprint or not.

Furthermore, in the event that the review includes data from more than 10 studies, ^46^ we will conduct univariate random-effects meta-regressions to investigate potential sources of variability in the prevalence of 1) data sharing (reported or actual availability) and 2) data availability statements by year of publication of primary studies, with bubble plots used to visualise regressions. If there are fewer than ten studies available to perform meta-regression, we will perform a subgroup analysis looking at differences in prevalence estimates across four time periods (Before 2010, 2010-2015, 2015-2020, 2020 onwards). These periods were chosen in order to isolate possible impacts of the COVID-19 pandemic (i.e., 2020 onwards) on prevalence rates, as well as investigate findings reporting an increase in uptake of data availability statements between 2014-2016. ^16^

### Sensitivity analysis

The team will perform four to five sensitivity analyses. First, we will conduct a sensitivity analysis to assess the robustness of pooled meta-analytic effect estimates based on the observed risk of bias of included studies. Specifically, we will compare pooled prevalence estimates of all studies eligible for meta-analysis against those rated as at a low risk of bias (refer to Assessment of the risk of bias in included studies for the risk of bias assessment). Second, we will conduct sensitivity analyses to examine whether estimates from studies not providing IPD differ from those where IPD were available, as well as whether estimates differ between studies that assessed availability in accordance with the FAIR principles to those that did not. Third, we will investigate differences in pooled prevalence rates when using logit-transformed proportions and generalized linear mixed models instead of Freeman-Tukey double arcsine transformations and standard inverse variance aggregation methods. ^47^ Lastly, the team may also perform sensitivity analyses on any set of two or more studies that include a large number of the same primary articles by removing the smallest affected studies from any relevant meta-analyses.

## Discussion

To our knowledge, this review will be the first study to estimate the prevalence of data and code sharing across the medical and health sciences. Our study will also use available IPD to investigate several aspects of data and code sharing that have not yet been well-explored, such as how sharing rates have changed over time, as well as what influence other relevant factors such as data and code availability statements and publishers’ and funders’ sharing policies have had on motivating medical and health researchers to share their data and code. Furthermore, appreciating regulatory changes which have further constrained international and intercontinental sharing of human research data, such as the introduction of the European Union’s General Data Protection Regulation in 2018, ^48^^,^^49^ we will also evaluate whether the type of research subjects studied impacts the likelihood of sharing. 

Our review has several strengths. First, the study will follow recommended practices in systematic review methodology by pre-registering the methods used to identify, select, and analyse eligible meta-research studies, and will declare any deviations from the protocol in the final publication. Furthermore, the review will systematically search multiple electronic databases for eligible articles, including preprint servers for unpublished work, as well as enlist at least two researchers to perform all article screening and data extraction tasks independently in parallel to minimise the chance of coding errors. The review will also share all data, materials and code generated by the study to allow others to verify or build upon our work.

However, there are also some limitations of this study. Importantly, this review will not place any strong restrictions on what constitutes ‘actual’ availability outside of requiring meta-researchers to have conducted some investigation into whether the data or code was indeed available. This is as opposed to requiring confirmation that data or code was shared in accordance with FAIR principles (i.e. is assigned aunique and persistent identifier, is associated with well-described meta-data and usage licenses, has been shared in a standardised format etc). ^50^ This decision was made based on our assumption that few potentially eligible meta-research studies will have assessed the FAIRness of data or code sharing, given familiarity with FAIR principles remains low. ^51^ Furthermore, given the novelty of the study, as well as appreciating that the establishment of metascience as a unique scholarly field is a relatively recent occurrence, there were few previous reviews, or universally agreed upon keywords and controlled vocabulary (e.g., MeSH and Emtree terms) with which to assist the search strategy development. Consequently, the lack of controlled vocabulary, as well as our limiting of searches to predominantly English-language databases may result in a greater risk of missing literature relevant to the research questions, when compared to other established review areas like reviews of randomised controlled trials where comprehensive guidance and established methodological search filters are available. ^52^ Furthermore, given the likelihood that IPD will not be available for all eligible meta-research studies, it is also possible that systematic biases may be present in the results of analyses reliant on IPD that will not be able to be detected.

## Conclusion

There is growing momentum among funders, publishers and the greater scientific community to increase the availability of the outputs of medical and health research. This review will provide some insight into how commonly data and code from medical and health research is shared. It will also examine how sharing rates have changed over time, and how influential some policies have been in motivating researchers to share their underlying data and code. It is expected that the findings of this research may be particularly useful to key research policymakers in developing, instituting and assessing policies on data and code sharing.

## Data and software availability

### Underlying data

No data are associated with this article. Data, materials and code from the completed review will be made freely available under a CC0 1.0 Universal license on the Open Science Framework ( https://osf.io/h75v4/).

### Extended data

Open Science Framework: A review of data and code sharing rates in medical and health research.

https://doi.org/10.17605/OSF.IO/H75V4. ^53^

This project contains the following extended data:

• Appendix-1.1_Search-Strategies-MedEmbMeta_v1.0.pdf (The proposed search strategy for MEDLINE, Embase and MetaArXiv)
• Appendix-1.2_Search-Strategies-MedBioRxiv_v1.0.R (The proposed search strategy for medRxiv and bioRxiv)
• Appendix-2_Data-Extraction-Fields_v1.0.pdf (The variables that will be extracted from eligible meta-research studies)

## Competing interests

DGH is a PhD candidate supported by an Australian Commonwealth Government Research Training Program Scholarship. The Laura and John Arnold Foundation funds the RIAT Support Centre (no grant number), which supports the salaries of ARF and KH. KH's project was supported by the Food and Drug Administration (FDA) of the U.S. Department of Health and Human Services (HHS) as part of a financial assistance award U01FD005946 totalling US$5,000 with 100 per cent funded by FDA/HHS. The contents are those of the author(s) and do not necessarily represent the official views of, nor an endorsement, by FDA/HHS, or the U.S. Government. The authors declare that no grants were involved in supporting this work.
