# Supplementary material for: Rates and predictors of data and code sharing in the medical and health sciences: Protocol for a systematic review and individual participant data meta-analysis
Source: F1000Res. 2021 Sep 9;10:491. Originally published 2021 Jun 22. [Version 2] doi: 10.12688/f1000research.53874.2 (PMC8485098; doi:10.12688/f1000research.53874.2)
Supplement: Peer review history

# Peer review: Reviewer response for version 2

**DOI:** 10.5256/f1000research.77267.r93873

**Published:** 2021-09-30

**Authors:** Jenine Harris

**Affiliations:** 1 Brown School, Washington University in St. Louis, St. Louis, MO, USA

The authors addressed my concerns thoroughly. The one point that I was thinking about re-addressing, the type of file data are shared in, may be moot anyway given the ability of open source software to import most types of data files.

 Nice work.

Is the study design appropriate for the research question?

Yes

Is the rationale for, and objectives of, the study clearly described?

Yes

Are sufficient details of the methods provided to allow replication by others?

Yes

Are the datasets clearly presented in a useable and accessible format?

Not applicable

Reviewer Expertise:

Increasing sex diversity in the STEM workforce; reproducible public health research

I confirm that I have read this submission and believe that I have an appropriate level of expertise to confirm that it is of an acceptable scientific standard.

# Peer review: Reviewer response for version 2

**DOI:** 10.5256/f1000research.77267.r93874

**Published:** 2021-09-09

**Authors:** Tim Hulsen

**Affiliations:** 1 Department of Hospital Services and Informatics, Philips Research, Eindhoven, The Netherlands

The authors responded well to all my remarks. I have no further comments.

Is the study design appropriate for the research question?

Yes

Is the rationale for, and objectives of, the study clearly described?

Yes

Are sufficient details of the methods provided to allow replication by others?

Yes

Are the datasets clearly presented in a useable and accessible format?

Not applicable

Reviewer Expertise:

Data management, data stewardship, data sharing, bioinformatics, data science.

I confirm that I have read this submission and believe that I have an appropriate level of expertise to confirm that it is of an acceptable scientific standard.

# Peer review: Reviewer response for version 1

**DOI:** 10.5256/f1000research.57299.r90452

**Published:** 2021-08-05

**Authors:** Jenine Harris

**Affiliations:** 1 Brown School, Washington University in St. Louis, St. Louis, MO, USA

This proposal describes a meta-analysis of studies of data and code sharing in medical and health sciences.

- There is a vast difference in the utility of available data uploaded via scanned pdf and available data in a csv file (or other software-neutral format), I would argue that csv data “available upon request” is more accessible than publicly available scanned pdfs containing data that will likely have to be re-entered by hand in order to be used. The authors might consider/measure data accessibility in addition to availability.
- Is there a reason for not confirming private availability in the same/similar way to public availability?
- Given the variety of search terms that could be used and non-standardized ways that journals operate and format manuscripts, would a secondary search strategy employing humans  potentially capture additional candidate studies?
- How will the authors handle data accessibility if the accessible data are synthetic? (E.g. when data cannot be made public due to private health info, some authors may choose to mimic important data features by providing synthetic data that will result in similar research results as the primary data)
- Will any attention be given to whether code that is available is in a format that can be read by open source software (e.g. Python, R) vs. making code available that can only be read with purchased software (e.g. SAS, SPSS)? Is code that someone needs to buy software to use really “open”?
- The secondary outcomes are all for the data sharing and not for the code sharing; would similar secondary outcome measures for code sharing be useful as well? Journals are unlikely to have code sharing policy (in my experience) but perhaps sharing of code increases with an open data policy. It seems logical that researchers who share data might be more inclined to share code as well.
- We found that researchers are more likely to share data and code when funders and employers require these things in public health (Harris *et al. *(2018 ^1^)). The authors are collecting funder policy on data (typo in data collection form lists “date” instead of “data”) but not employer. Adding employer may provide some additional insight into why certain papers have available data/code.

Is the study design appropriate for the research question?

Yes

Is the rationale for, and objectives of, the study clearly described?

Yes

Are sufficient details of the methods provided to allow replication by others?

Yes

Are the datasets clearly presented in a useable and accessible format?

Not applicable

Reviewer Expertise:

Increasing sex diversity in the STEM workforce; reproducible public health research

I confirm that I have read this submission and believe that I have an appropriate level of expertise to confirm that it is of an acceptable scientific standard, however I have significant reservations, as outlined above.

## References

[1] : Use of reproducible research practices in public health: A survey of public health analysts.*PLoS One*.2018;13(9) : 10.1371/journal.pone.0202447e020244710.1371/journal.pone.020244730208041PMC6135378

# Response

**Published:** 2021-08-31

**Authors:** Daniel Hamilton

**Affiliations:** University of Melbourne, Australia

Thank you very much for the useful feedback Jenine. Please see our responses to your comments in a point-by-point manner below.

- *There is a vast difference in the utility of available data uploaded via scanned pdf and available data in a csv file (or other software-neutral format), I would argue that csv data “available upon request” is more accessible than publicly available scanned pdfs containing data that will likely have to be re-entered by hand in order to be used. The authors might consider/measure data accessibility in addition to availability.*
- **Response:** We agree that data shared in machine-readable formats are much more valuable than data shared in non-machine-readable formats - this notion being a key element of the ‘Interoperability’ criterion of the FAIR principles. This comment, as well as the second comment from Reviewer #1, has motivated us to add some text to outline this study limitation (Discussion: Paragraph 3, Sentences 2-4), record more detailed information on how meta-research studies define ‘actual availability’, as well as include an extra sensitivity analysis to estimate the prevalence of FAIR sharing of data and code, and how these estimates compare with the ‘actual availability’ estimates (Methods/Sensitivity analysis: Paragraph 1, Sentence 4).
- *Is there a reason for not confirming private availability in the same/similar way to public availability?*
- **Response:** This is a great point. This was an oversight. In response, we have brought the private availability outcome measures in line with the public availability outcome measures (refer to Methods/Types of methods and Methods/Types of outcome measures).
- *Given the variety of search terms that could be used and non-standardized ways that journals operate and format manuscripts, would a secondary search strategy employing humans potentially capture additional candidate studies?*
- **Response:** Given the terms used to describe open practices such as data and code sharing vary substantially among manuscripts, we plan to screen reference lists of relevant studies identified by the search, as well as the bibliographies of all included studies. We will also contact the authors of relevant studies and others with expertise in this area, conduct forward citation searches of included articles, as well as browse other preprint servers and online resources (Open Science Framework, aspredicted.org, connectedpapers.com) to help identify further studies.
- *How will the authors handle data accessibility if the accessible data are synthetic? (E.g. when data cannot be made public due to private health info, some authors may choose to mimic important data features by providing synthetic data that will result in similar research results as the primary data).*
- **Response:** This is a very interesting point. Firstly, for the purposes of the review we will not exclude meta-research studies if they do or don't include data from *in silico *experiments within their scope. Secondly, we will also not exclude meta-research studies if they: 1) considered the sharing of synthetic data as acceptable or not, or 2) generated a synthetic dataset for the purposes of sharing the data from their own study. While we do not expect any of the eligible meta-research studies to have come across synthetic data when assessing data availability, nor used this approach when sharing their own data given it appears to be still in its infancy (10.7554/eLife.53275), some text has been added to the methods to clarify the above points (Methods/Types of Studies: Paragraph 2, Sentence 1).
- *Will any attention be given to whether code that is available is in a format that can be read by open source software (e.g. Python, R) vs. making code available that can only be read with purchased software (e.g. SAS, SPSS)? Is code that someone needs to buy software to use really “open”?*
- **Response: **We do not plan to differentiate between sharing code for proprietary or open-source software for the reasons discussed in Comment #1. As long as it can, in principle, be scrutinised it will be considered open for the purposes of the review. This will be a limitation that will be discussed in the final paper, along with other FAIR principles considerations, and the findings of the FAIRness sensitivity analysis.
- *The secondary outcomes are all for the data sharing and not for the code sharing; would similar secondary outcome measures for code sharing be useful as well? Journals are unlikely to have code sharing policy (in my experience) but perhaps sharing of code increases with an open data policy. It seems logical that researchers who share data might be more inclined to share code as well.*
- **Response:** Another great point. We agree that researchers who share data may be more inclined to share their code too and so have added this as an extra secondary outcome measure. We have also used this opportunity to add in two further secondary outcome measures to capture the overall prevalence of code availability statements, as well as their potential impact in incentivising researchers to share their code publicly (refer to Methods/Types of outcome measures).
- *We found that researchers are more likely to share data and code when funders and employers require these things in public health (Harris et al. (2018)). The authors are collecting funder policy on data (typo in data collection form lists “date” instead of “data”) but not employer. Adding employer may provide some additional insight into why certain papers have available data/code.*
- **Response:** Thank you for another great point. We have added this as another variable to collect and corrected the flagged typographical error (refer to version 2 of Appendix 2 on our OSF page).

# Peer review: Reviewer response for version 1

**DOI:** 10.5256/f1000research.57299.r88792

**Published:** 2021-07-02

**Authors:** Tim Hulsen

**Affiliations:** 1 Department of Hospital Services and Informatics, Philips Research, Eindhoven, The Netherlands

The manuscript presents a protocol on the study of sharing data and source code in the medical domain. The methodology that is described in the paper seems scientifically sound. I do have some suggestions to improve the paper:

- When discussing data sharing, it is important to note the existence of privacy laws such as the GDPR (EU), and HIPAA (USA), since they have such a large effect on data sharing practices (e.g. obligatory data management plans, data controller/processor definition, detailed informed consents, etc.)
- Data sharing is a first step, but the data should also be made available in a way that it can be reused. I was surprised that the FAIR (Findability, Accessibility, Interoperability, Reusability) principles are not mentioned in the paper. Shared datasets (or code) that adhere to these guidelines are much more likely to be reused. See Wilkinson *et al.* (2016 ^1^).
- Selection of studies: "We will attempt to translate foreign-language articles flagged as potentially eligible using Google Translate or native speakers known to the team. If unable to translate the document successfully, we will exclude the study." Doesn't this include a bias in your study? Only if the article is written in a language known to people in your environment, or in a language that can be translated well by Google Translate, it is included.

Is the study design appropriate for the research question?

Yes

Is the rationale for, and objectives of, the study clearly described?

Yes

Are sufficient details of the methods provided to allow replication by others?

Yes

Are the datasets clearly presented in a useable and accessible format?

Not applicable

Reviewer Expertise:

Data management, data stewardship, data sharing, bioinformatics, data science.

I confirm that I have read this submission and believe that I have an appropriate level of expertise to confirm that it is of an acceptable scientific standard, however I have significant reservations, as outlined above.

## References

[1] : The FAIR Guiding Principles for scientific data management and stewardship.*Sci Data*.2016;3: 10.1038/sdata.2016.1816001810.1038/sdata.2016.1826978244PMC4792175

# Response

**Published:** 2021-08-31

**Authors:** Daniel Hamilton

**Affiliations:** University of Melbourne, Australia

Thank you very much for the useful feedback Tim. Please see our responses to your comments in a point-by-point manner below.

- *When discussing data sharing, it is important to note the existence of privacy laws such as the GDPR (EU), and HIPAA (USA), since they have such a large effect on data sharing practices (e.g. obligatory data management plans, data controller/processor definition, detailed informed consents, etc.)*
- **Response: **This is a very pertinent point. In response, we have added some text to the discussion concerning the impact of the recent regulatory changes on data sharing behaviours - particularly sharing health data derived from human research participants across international and intercontinental borders (refer to Discussion: Paragraph 1, Sentence 3).
- *Data sharing is a first step, but the data should also be made available in a way that it can be reused. I was surprised that the FAIR (Findability, Accessibility, Interoperability, Reusability) principles are not mentioned in the paper. Shared datasets (or code) that adhere to these guidelines are much more likely to be reused. See Wilkinson et al. (2016)*.
- **Response:** We agree that when sharing data or code it should comply with the FAIR principles. The principles were certainly considered when developing the protocol, however there are a couple of reasons why we decided not to require studies to have strictly assessed the ‘FAIRness’ of shared data or code to be eligible for inclusion in the review. Firstly, given that the FAIR principles were drafted in 2015, any relevant literature published prior to this date would have to be excluded. Secondly, pilot searching demonstrated that there are likely very few (possibly no) meta-research studies conducted after 2015 that looked at data or code availability judged according to the FAIR principles (i.e. data or code were only considered ‘available’ if it met all FAIR criteria). This perhaps is not surprising as familiarity with FAIR principles remains low (10.6084/m9.figshare.13274744). Despite this, we have attempted to begin to bridge this gap via our ‘actual availability’ measure, which requires meta-researchers to have conducted some investigation into whether the data or code is indeed available (i.e. akin to assessing the ‘F’ in FAIR). However, given we suspect this will be a limitation of the majority of included studies, it will therefore also be a limitation of the review. Ultimately, we agree that not mentioning the principles in the discussion is an oversight and so have added some text (refer to Discussion: Paragraph 3, Sentences 2-4). We will also record the methods each meta-research study used to define ‘actual data availability’ and if any meta-research studies stipulated that they required shared data or code to comply with the FAIR principles in order to be considered ‘available’, we will also present a synthesis of such studies in the form of a sensitivity analysis (refer to Methods/Sensitivity analysis: Paragraph 1, Sentence 4). 
- *Selection of studies: "We will attempt to translate foreign-language articles flagged as potentially eligible using Google Translate or native speakers known to the team. If unable to translate the document successfully, we will exclude the study." Doesn't this include a bias in your study? Only if the article is written in a language known to people in your environment, or in a language that can be translated well by Google Translate, it is included.*
- **Response:** You are correct. Excluding foreign-language studies that cannot be successfully translated could unfortunately indeed introduce a bias. Recognising language bias to a greater or lesser extent is present in most systematic reviews (10.11124/JBIES-20-00361) we have taken steps to minimise this by attempting to translate articles in languages other than English, and note that Google Translate offers translation for over 100 languages. Further, any concerns we have around the exclusion of potentially relevant articles based on language will be addressed as a limitation of the review. 
